# Evaluating mHealth Applications for Older Adults with Hypertension: Systematic Meta-Analysis of Clinical Trials

**DOI:** 10.1093/geroni/igaf122.517

**Published:** 2025-12-31

**Authors:** Renato Ferreira, Leitao Azevedo, Michael Varzino, Nidhi Patel, Wendy Rogers

**Affiliations:** University of Georgia, Athens, Georgia, United States; University of Illinois Urbana-Champaign, Champaign, Illinois, United States; University of Illinois Urbana-Champaign, Chicago, Illinois, United States; University of Illinois Urbana-Champaign, Champaign, Illinois, United States

## Abstract

Hypertension is the most prevalent chronic condition affecting older adults and mobile health (mHealth) applications have the potential to support their blood pressure (BP) management and self-care. However, no meta-analysis examines the effects of mHealth app interventions for older adults. To address this gap, we conducted a meta-analysis of randomized controlled trials (RCTs) to examine the effectiveness of mHealth apps for improving older adults’ hypertension outcomes. Relevant studies were sought through EBSCO, Clinical Trials, Cochrane Library, ProQuest, PubMed/MEDLINE, ScienceDirect, Scopus, and Web of Science databases. We retrieved 1,244 papers and two reviewers completed a full screen review of 79. We excluded studies that were not RCTs, did not examine mHealth apps effects, or were not conducted with older adults, resulting in 9 papers with a total of 915 participants included in the analysis. Our results indicate that mHealth apps can be effective for older adults managing hypertension, with significant reductions in Systolic BP (Hedge’s g: -1.37, 95% CI [-2.25 to -0.49]), and Diastolic BP levels (g: -079, 95% CI [-1.42 to -0.16]), as well as signs of improvement in medication adherence (g: 3.70, 95% CI [-29.82 to 37.22]). These effects are consistent with those meta-analysis involving other age groups, supporting that older adults can benefit from mHealth apps. We review each intervention, assess potential biases and the app components that may impact effectiveness. This analysis aims to guide the design of future hypertension management RCTs, and more broadly, improve the design of other mHealth tools for older adults. Pre-registration: https://doi.org/10.17605/OSF.IO/NS7TP

